# Red shell‐ high risk normal tissue in stereotactic radiosurgery

**DOI:** 10.1002/pro6.1218

**Published:** 2023-12-25

**Authors:** Jun Yang, Weihua Qi, Lei Wang, Qiuxia Lu, Liangfu Han, Brian Wang, Weisi Yan

**Affiliations:** ^1^ Junxin Precision Oncology Group Foshan Guangdong China; ^2^ Radiation Oncology Dept Foshan Chancheng Hospital Foshan Guangdong China; ^3^ Baptist healthcare system Kentucky USA

**Keywords:** biological equivalent dose, normal tissue, Red Shell, stereotactic radiosurgery, toxicity

## Abstract

Due to the ablative nature of high prescription in Stereotactic Radiosurgery or stereotactic body radiation therapy (SRS/SBRT), the normal tissue surrounding the CTV receives the dose higher than tissue's dose constraint. A concept of Red Shell is proposed to define and quantify these tissue damaged in SBRT, using biological equivalent dose (BED) concept. The combination of biological factors and physics factors, including serial and parallel organ, dose gradient, dose distribution and fractionations, are further discussed to interpret the clinical meaning of Red Shell. This concept can also help planner to improve the optimization in planning process.

## Graphical abstract

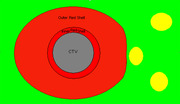

## 1 INTRODUCTION OF RADIOSURGERY

Traditional stereotactic radiosurgery (SRS), such as Cobalt based Gamma knife and linac based X knife, delivers high radiation dose to the intracranial targets in single fraction. The treatment dose could vary from 10 to 24 Gy depending on the type and size of tumors. New development of the radiosurgery equipment, like Cyberknife or other advanced linear accelerator, expands the radiosurgery technique to treat extracrianial tumors and the treatments are extended up to 5 fractions. The ablative dose scheme of hypofractioned radiosurgery is more sophisticated and the radiobiology of hypofractioned radiosurgery to extracranial target is a relative new area to the radiation oncology.^1^

Compared with the 2 Gy per fraction for 25–35 fractions in conventional radiation therapy, the radiosurgery delivers a much higher dose each fraction and less fractions are applied. The total biological equivalent dose (BED) is usually higher than the conventional radiation therapy.^2^

Many radiation beams (or arcs) crossed in the target and delivers high radiation to the target. Because of the big number of beams, each beam delivers relative low dose and it is not too strong to damage the normal tissue passing through. Since radiosurgery treatment field size is small and some of radiosurgery systems do not have flattening filter, the dose in the center of target is higher than the dose in the boundary of target. The dose falls off fast outside of target with the distance to the target.

The treatment delivery precision is crucial in radiosurgery. There are two different methods to guarantee the sub‐millimeter treatment precision. The first is to use mechanic apparatus, like head frame etc to rigidly immobilize the patient. The target is fixed at the treatment iso‐center, and radiation sources surrounding the target deliver radiation precisely to the target. The second method is to allow target to have limited movement, machine tracks the target and corrects the displacement before turning on radiation.

## 2 RADIOBIOLOGY EQUIVALENT DOSE

The linear quadratic (LQ) model ^3^, ^4^, ^5^ was used to calculate the BED of hypofractionated radiosurgery as

$$\mathrm{BED}\;=\frac{E}{\alpha }\;=\;nd\left(1+\frac{d}{\alpha /\beta }\right)$$

where *n* is the number of fractions, *d* is the dose per fraction in Gy, *α* represents the linear component of cell killing, and *β* represents the quadratic component of cell killing. and *α/β* is determined from cell survival curves or in vivo data. *α/β* is about 10 Gy for early response tissues and about 3 Gy for late response tissues.

Conventional radiation therapy reports severe normal tissue complications with normalized total dose (NTD) of 70 Gy (2 Gy per fraction) which has the BED of 117 Gy_3_. The different doses of fractioned radiosurgery (1 to 6 fractions) with BED of 117 Gy_3_ have been calculated, and the results are summarized in Table 1.

**TABLE 1:** Fraction scheme generating BED of 117 Gy_3._

	Fx = 1	Fx = 2	Fx = 3	Fx = 4	Fx = 5	Fx = 6	Fx = 35
Dose/Fx (Gy)	17.3	11.8	9.4	8.0	7.0	6.3	2.0
Total Dose (Gy)	17.3	23.6	28.2	32	35	37.8	70

## 3 RED SHELL

### 3.1 The Concept and Definition of Red Shell

Since the prescription dose delivered to the target is high, the adjacent normal tissue receives relatively high dose inevitably. Due to the dose fall‐off, the closer tissue is to the target, the higher dose the tissue receives. Because of the high dose gradient in radiosurgery, the tissue that is further from target could tolerant the treatment well. Only the normal tissue that is very close to the target gets high radiation dose and needs to be scrutinized.

**FIGURE 1:**
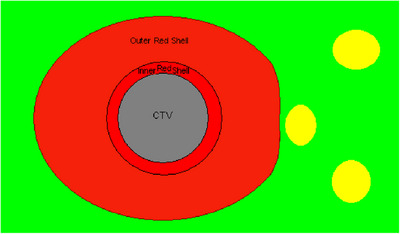
The Red Shell shows in red, surrounding the CTV in green. The inner boundary of Red Shell is the CTV outer surface, and the outer boundary is where the BED drops to tissue's constraint dose.

We define “Red Shell” as the tissue between the border of clinical target volume (CTV) and a lower dose level that we can be sure will not harm locally the normal tissues around the CTV.^6^, ^7^ (Figures 1) A zone that goes all the way around the CTV between whatever the prescription dose is and a “constraint dose” which could be assumed to be the biological equivalent of an NTD of 70 Gy (in 2 Gy fractions) = a BED = 117 Gy_3_. If for 3 fractions it would be still BED = 117 Gy_3_; now = 9.3 Gy in 3 fractions; or if for 5 fractions still BED = 117 Gy_3_; but then = 7 Gy in 5 fractions. This would be a Red Shell of probably a few mm thickness. The inner surface of the shell, at the CTV, would be set by the CTV; the outer surface would be set by that BED equivalent of 70 Gy NTD for the whole treatment. It is specially designed for the large individual fractions in Stereo‐type set‐ups. If the prescription dose were exactly 7 Gy ×5 F, then the thickness of the Red Shell would be planning target volume (PTV) margin. The same applies for lower doses per fraction.

**FIGURE 2:**
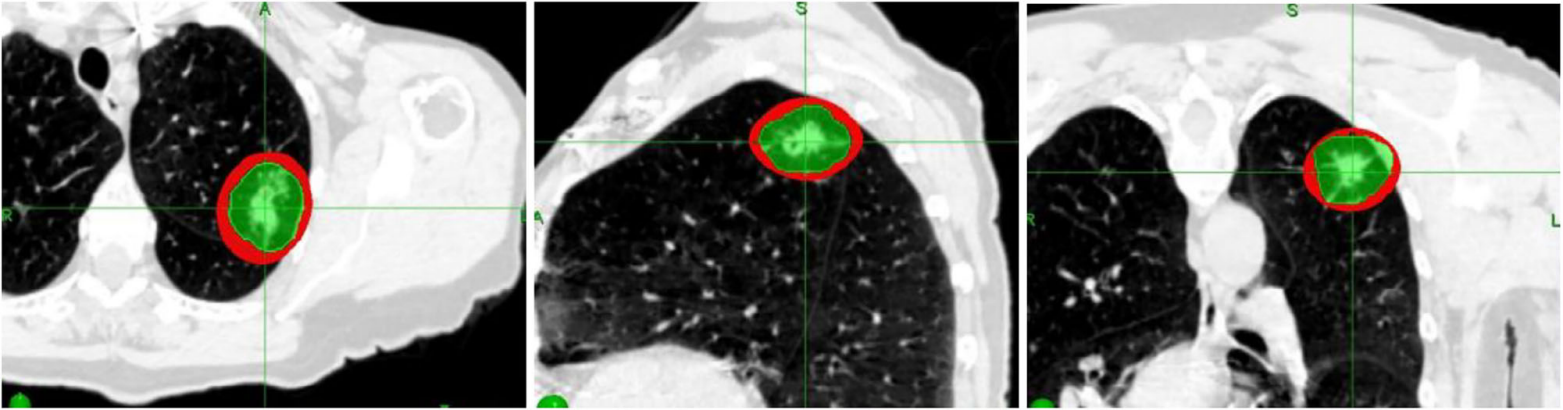
The Red Shell showed in axial, sagittal and coronal views of a clinical lung SRS case. The thin Red Shell surrounds the green CTV.

At the outer surface of the Red Shell, the chances of any local damage to a normal tissue structure situated there would be very small (≤ 70 Gy NTD) and we could say “Good chance of Safe”. One would not worry too much about a normal tissue structure sitting there. However, at the inner surface of the Red Shell, the BED and total dose (TD) would be set by the prescription dose, which might be very high indeed, so no normal tissue would survive there. This would be prohibitively high for example with 3F × 18–20 Gy. Which is BED = 375–460 Gy_3_; = NTD = 227–276 Gy in 2 Gy Fr's for the whole treatment. Many mm distance away would NTD = 70 Gy come, before we could think “Good chance of safe”. Figures 2 is a clinical example of Red Shell of a lung SBRT/SrS.

The characteristics of the Red Shell are: the volume and the thickness. The thickness could/wound be different in different directions depending on the treatment planning. We denote the Red Shell as:

RS(Prescribed Dose/Normal Toleration. X fractions) = X mm or
RS(Prescribed Dose/Normal Toleration. X fractions) = X cc

The *volume* of the Red Shell is decided by many factors, such as the prescription dose, PTV size, dose gradient, number of fractions, and the technique used in the treatment. In addition, the radiosensitivity and type of tissues at risk, including their *α/β* ratios, lead to an estimate of the constraint NTD contour. The *shape* of the Red Shell is dependent on the shape of the PTV and, after some preliminary planning, the proximity of critical organs.

### 3.2 Clinical interpretation of red shell concept

As the Red Shell represents the normal tissue under high risk of radiation damage in SRS or stereotactic body radiation therapy (SBRT), the display of Red Shell helps the clinician to pay extra attention to the tissue that is near CTV. Red shell also helps planner to quantify the organ at risk and improve the quality of plan accordingly.

#### 3.2.1 Parallel organ and serial organ

There is various types of tissue around the target. In the above descriptions, to help simplify the discussion, we have not deviated the constraint value for the threshold “significant late risk” from an NTD 70 Gy. That is, a late BED of 117 Gy_3_. (This is also a tumor BED of 84 Gy_10_, for most tumors except prostate, malignant melanoma, and breast cancer). It is entirely possible that the tissues at risk might be more radiosensitive than we have assumed here, such as, an NTD of 50, 24 or 20 Gy in 2 Gy fractions.

In practice, parallel organ and serial organ are evaluated differently in Red Shell. Most of serial organs can tolerate NTD 70 Gy as normal tissue but only to a very small volume without breaking down the organ's function. The planer should try all means to avoid Red Shell overlapping with serial organs, such as bowel, cord, optical chiasm etc, which often need to compromise dose conformality or dose gradient to protect the serial organ.

The tissue in typical parallel organ, such as lung, liver or brain tissue, generally have lower dose tolerance (in the range of NTD 20–54 Gy) and the remaining tissue of the parallel organ outside of Red Shell is still working to keep the whole organ functional. However, the volume of parallel organ covered by the Red Shell should be minimized in order to best protect the reserves of parallel organs. This has often been overlooked as conventional dose constrains are typically serial organ focused. In SRS/SBRT era, multiple treatment courses can be applied to patient's same parallel organ (multiple brain mets for example) in years and it is critical to minimize the amount of parallel tissue damage in each course so that patient can have enough reserves to tolerate multiple courses.

#### 3.2.2 Dose gradient

In order to minimize the thickness or the volume of Red Shell, high dose gradient outside of PTV is highly desired. The 10% dose outside of PTV fall off every 0.5‐2 mm is a common gradient in radiosurgery. In the following picture, the left image is the dosimetric plan treating tumor with 7 photon beams, the middle image is that of Tomotherapy/RapidARC type of single plane treatment, and the right image is the Cyberknife plan with many non‐coplanar beams. It is clear that Tomothearpy and Cyberknife plan are superior to 7‐beam plan, since a fairly large number of beams (>80) are used and each beam carries relative smaller percentage of machine unit. Each beam gives little dose to the peripheral normal tissue that it passes through. When many beams intersect at the PTV, the dose to the tumor is high and the dose falls off fast outside of PTV.

**FIGURE 3:**
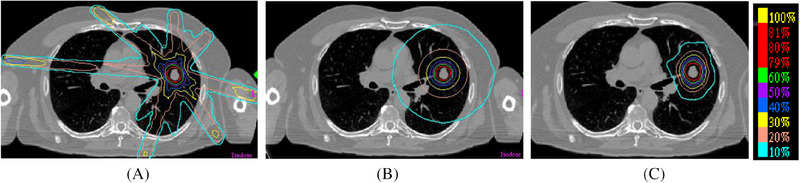
Dose gradients of coplanar and non‐coplanar SRS. (A) 7‐Beam SRS; (B) Single Plane SRS; (C) Non‐coplanar SRS

When a single plane SRS is compared with non‐coplanar SRS, the dose gradient of non‐coplanar SRS is higher since the dose is spread out from a wider solid angle (Figure 3). To achieve a high dose gradient, many beams from different angles (non‐coplanar beam) are preferred.

#### 3.2.3 The Isotropic VS Anisotropic dose distribution

If target locates in the parallel organ, like lung, liver and brain, we would like the dose distributes evenly around the PTV. The dose isotropy is determined by dose distribution of treatment plan, and particularly the angle the photon beams irradiating from and the weight the beams carry. If the equal weighted beams irradiating the target distributed evenly in the solid angle, the dosimetric plan has higher and better isotropy.

**FIGURE 4:**
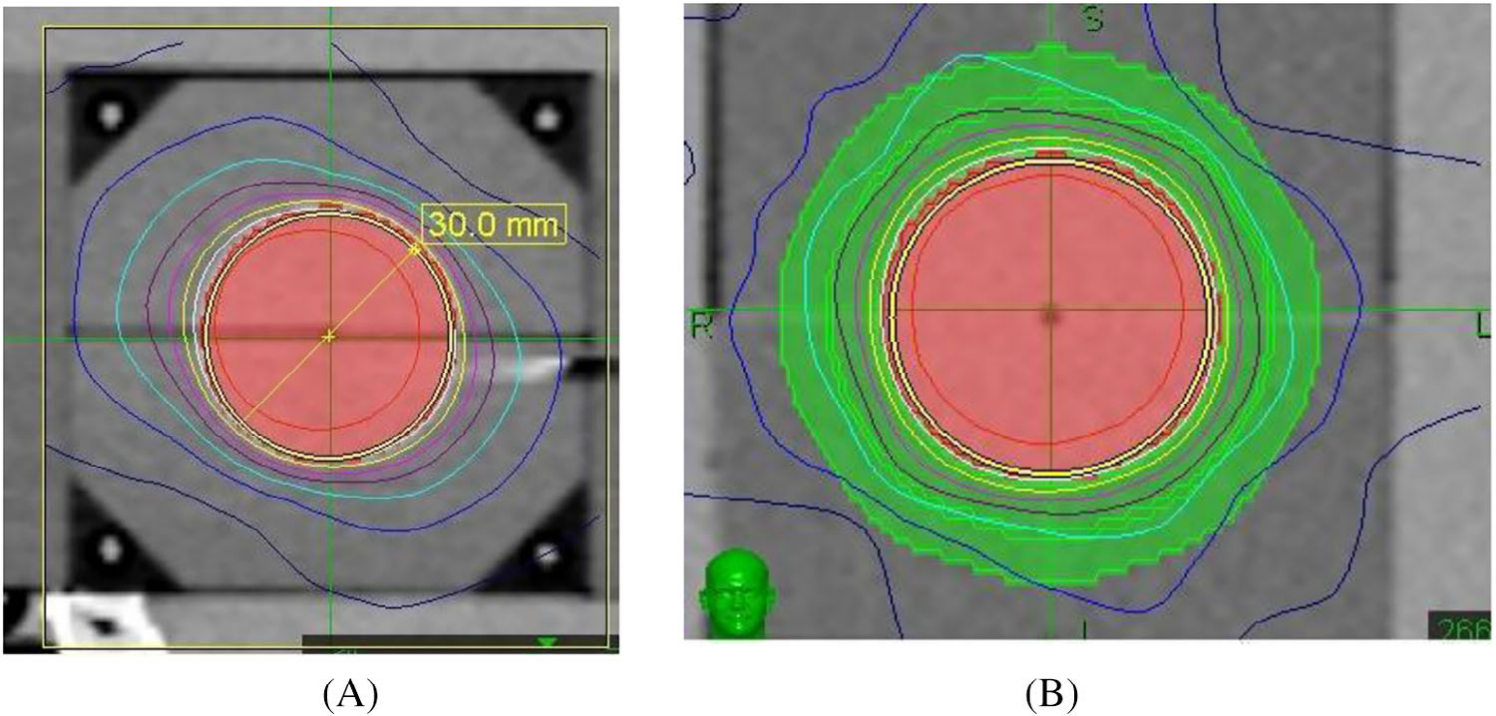
Isotropic dose distribution of Cyberknife. (A) The Shell will be slightly thicker in the orientation where beams come from. (B) Cyberknife can adjust each beam's weight, and make the shell more equally thick.

In the simplified hypothetical situation: a tumor with sphere shape, and it is desired that the red shell also has sphere shape. It may only happen with some tumor treated with Gamma knife, as the evenly (Not perfectly even though) distributed 201 cobalt sources deliver same amount of radiation. Since Cyberknife dose comes from most anteriorly and laterally, but rarely posteriorly. The Shell will be slightly thicker in the orientation where beams come from (Figure 4a). The advantage of Cyberknife is that it can adjust each beam's weight, and make the dose more isotropic, hence make the shell more equally thick (Figure 4b). Tomotherapy's shell in this situation, will be equally thick in axial plane, but will be much thinner in Superior‐Inferior direction since Tomotherapy only deliver radiation within a single plane. When the tumor's shape is irregular, the anisotropy will become more obvious.

#### 3.2.4 Fractionations

How can we minimize the volume of Red Shell? We can either do a best plan in order to increase the dose gradient, which will reach certain physics limitation. In addition, we can consider change the fractionation schemes, to decrease the BED to the normal tissue.^8^

For example, in a lung case, we can prescribe either 20 Gy ×3 F or 14.1 Gy ×5 F with the same BED of 120 Gy_20_ to the tumor. The physical dose distributions and dose gradient are same, except the dose prescription are different. The Red Shell of both prescription schemes are calculated as following, assuming the tissue's dose constraint is BED 117Gy_3_(Table 2).

**TABLE 2:** The Red Shell of two prescription schemes (assuming the tissue's dose constraint is BED 117Gy_3_).

	Plan I	Plan II
Prescription dose	20 Gy ×3 F	14.1 Gy ×5 F
BED(Gy_20_) to Tumor	120 Gy_20_	120 Gy_20_
Tissue Constrains dose BED 117Gy_3_	9.4 Gy ×3 F	7 Gy ×5 F
The ratio of dose with BED 117Gy_3_ to Prescription Dose	= (9.4×3)/(20×3) = 47.0%	= (7×5)/(14.1×3) = 49.7%

**FIGURE 5:**
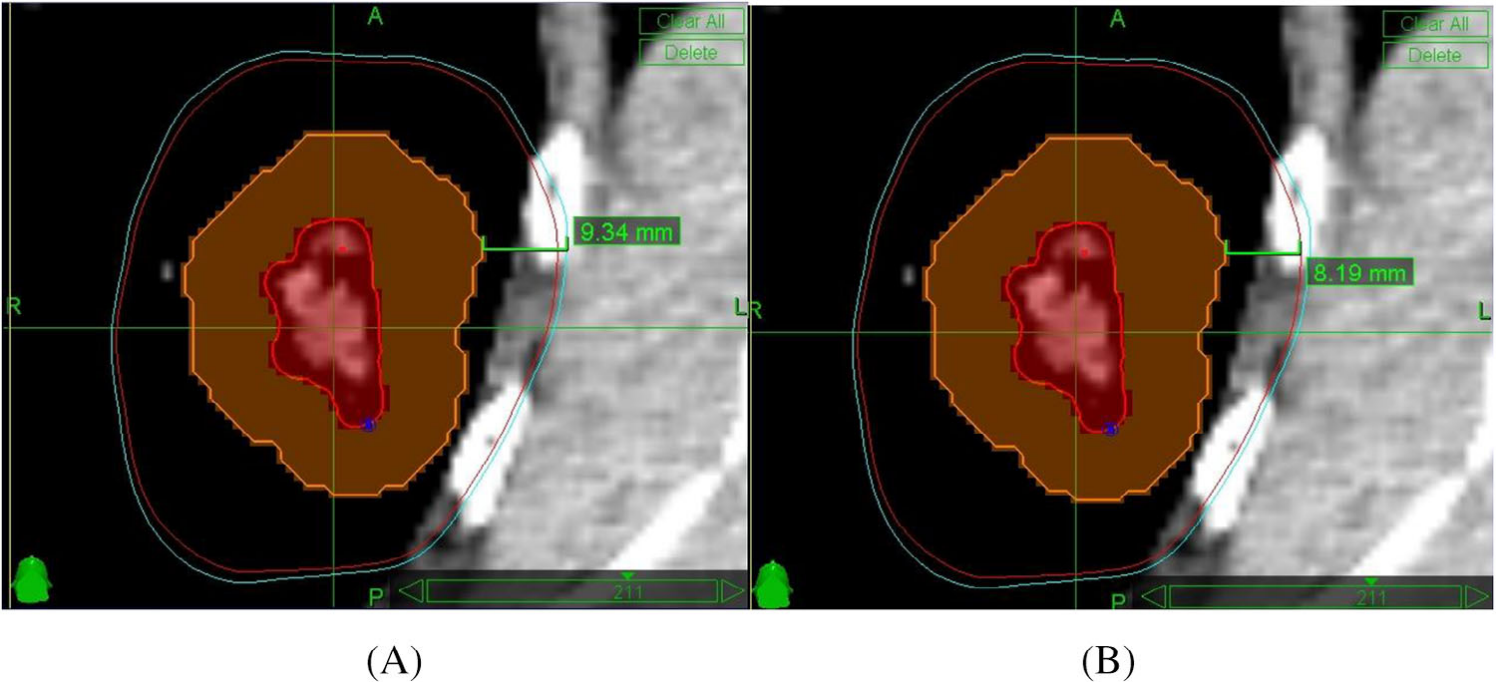
A lung SBRT plan where the GTV is contoured in thick red and the PTV is contoured in the organ line. The blue line is 47.0% iso‐dose (A) representing the tissue dose constrain in 20 Gy ×3 F scheme and the red line is 49.7% iso‐dose (B) b representing the tissue dose constrain in 14.1 Gy ×5 F scheme.

In plan I, any tissue receiving 47.0% of prescription dose is covered by the Red shell(Figure 5a). In plan II, the tissue receiving 49.7% of prescription dose is covered by the Red shell (Figure 5b).

As shown in the following images of lung SBRT case, the Shell thickness decrease from 9.34 mm in 20 Gy ×3 F scheme to 8.19 mm in 14.1 Gy ×5 F scheme, and the shell volume drops from 37.4 cc to 32.8 cc.

## 4 DISCUSSION

The tissue damage of SBRT has always received attention due to the ablative dose and short treatment course. We have built the relationship between the risk to normal tissue damage and the dose schemes, and type of normal tissue treated. We presented a new concept of Red Shell in SBRT to denote the region between the CTV and constrained healthy tissue dose. The tissue that received a non‐negligible, possibly high, risk of radiation damage is defined and quantified as SBRT.

In addition, the safety of a SBRT plan can be evaluated by checking the volume of tissue either serial type or parallel type covered by a Red Shell. We suggest that Red Shell should be adopted as one of the parameters in SBRT clinical planning process, and the treatment planning system should report the Red Shell in the dosimetric report display. The Red Shell couldalso be part of the objective function of planning optimization, so that minimizing the Red Shell damage could be part of the optimization as the “cost” of treatment.

## CONFLICT OF INTEREST STATEMENT

The authors declare that there are no competing interests.

## ETHICS STATEMENT

Not applicable.
